# Headspace SPME GC–MS Analysis of Urinary Volatile Organic Compounds (VOCs) for Classification Under Sample-Limited Conditions

**DOI:** 10.3390/metabo16010057

**Published:** 2026-01-08

**Authors:** Lea Woyciechowski, Tushar H. More, Sabine Kaltenhäuser, Sebastian Meller, Karolina Zacharias, Friederike Twele, Alexandra Dopfer-Jablonka, Tobias Welte, Thomas Illig, Georg M. N. Behrens, Holger A. Volk, Karsten Hiller

**Affiliations:** 1 Department of Bioinformatics and Biochemistry, BRICS—Braunschweig Integrated Centre of Systems Biology, Technische Universität Braunschweig, 38106 Braunschweig, Germany; l.woyciechowski@tu-braunschweig.de (L.W.);; 2 Department of Small Animal Medicine and Surgery, University of Veterinary Medicine Hannover, 30559 Hannover, Germany; 3 Center for Systems Neuroscience Hannover, 30559 Hannover, Germany; 4 Department of Rheumatology and Immunology, Hannover Medical School, 30625 Hannover, Germany; 5 Department of Respiratory Medicine and Infectious Diseases, Hannover Medical School, 30625 Hannover, Germany; 6 Hannover Unified Biobank (HUB), Hannover Medical School, 30625 Hannover, Germany

**Keywords:** HS–SPME, GC–MS, urinary VOCs, urine, volatilomics, non-targeted profiling, classification, small sample volume, pH adjustment, CAR/PDMS fiber

## Abstract

Background/Objectives: Volatile organic compounds (VOCs) are emerging as non-invasive biomarkers of metabolic and disease-related processes, yet their reliable detection from complex biological matrices such as urine remains analytically challenging. This study aimed to establish a robust, non-targeted headspace solid-phase microextraction gas chromatography–mass spectrometry (HS–SPME GC–MS) workflow optimized for very small-volume urinary samples. Methods: We systematically evaluated the effects of pH adjustment and NaCl addition on VOC extraction efficiency using a 75 µm CAR/PDMS fiber and a sample volume of only 0.75 mL. Method performance was further assessed using concentration-dependent experiments with representative VOC standards and by application to real human urine samples analyzed in technical triplicates. Results: Acidification to pH 3 markedly improved extraction performance, increasing both total signal intensity and the number of detectable VOCs, whereas alkaline conditions and additional NaCl produced only minor effects. Representative VOC standards showed compound-specific linear dynamic ranges with minimal carry-over within the relevant analytical range. Application to real urine samples confirmed high analytical reproducibility, with triplicates clustering tightly in principal component analysis and most metabolites exhibiting relative standard deviations below 25%. Conclusions: The optimized HS–SPME GC–MS method enables comprehensive, non-targeted urinary VOC profiling from limited sample volumes. This workflow provides a robust analytical foundation for exploratory volatilomics studies under sample-limited conditions and supports subsequent targeted method refinement once specific compounds or chemical classes have been prioritized.

## 1. Introduction

Volatile organic compounds (VOCs) provide a unique, non-invasive fingerprint of metabolic activity, yet their reliable detection and interpretation remain analytically challenging. These small molecules (typically <500 Da) are characterized by high vapor pressure and low boiling points. They can originate from exogenous sources, such as diet and pharmaceuticals, or be produced endogenously as end products of carbohydrate and lipid metabolism, microbial fermentation in the gut, or oxidative stress [1,2]. Once formed, VOCs can enter the bloodstream and be excreted via urine, sweat, breath, or saliva [1], enabling minimally invasive detection. Because they reflect underlying metabolic processes, VOC profiles have been increasingly investigated as potential disease biomarkers [3,4]. Disease-specific VOC signatures have been described in conditions such as cancer, metabolic disorders, and infectious diseases [5,6,7]. Beyond clinical diagnostics, VOC analysis has also been applied in pharmacological and toxicological studies, including the detection of psychoactive substances [8].

Among the biological matrices available for volatilomics, urine is particularly attractive. It is collected non-invasively, reflects systemic metabolism, and has already been reported to contain more than 400 distinct VOCs spanning chemical classes such as hydrocarbons, aldehydes, ketones, organic acids, alcohols, and phenols [9]. Several studies and reviews have summarised urinary VOCs as potential biomarkers for cancer and other diseases, highlighting both the promise and the current lack of standardisation in analytical protocols [4]. In addition, a recent review in Biomedicines has specifically discussed urinary VOCs for non-invasive tumour detection and emphasised the heterogeneity of reported biomarkers and methods and the need for robust, clinically applicable workflows [10]. Against this background, our study focuses on the development and validation of a small-volume, non-targeted HS–SPME GC–MS protocol suitable for biobank and clinical applications. However, urine is also a chemically complex and variable fluid, containing metabolic waste, diet-derived molecules, bacterial by-products, and environmental contaminants. This complexity, together with urine’s high water content, makes reliable extraction of VOCs technically demanding. To improve sensitivity, pre-concentration from the sample headspace (HS) is typically required. A widely adopted technique is solid-phase microextraction (SPME), which integrates extraction and pre-concentration into a single solvent-free step. Since its introduction in the 1990s, HS–SPME has become popular for VOC analysis due to its simplicity, cost-effectiveness, and broad applicability [11].

Despite widespread use, HS–SPME protocols for urinary VOCs vary considerably [12]. Urine’s pH, ionic strength, and storage conditions strongly influence VOC partitioning into the HS, thereby affecting extraction efficiency [2,3,13]. Acidification has been shown to enhance the recovery of acidic analytes, while alkaline adjustment yields more for the extraction of basic analytes [14].

Similarly, salt addition can promote the salting-out effect, further shifting VOCs from the aqueous phase into the HS. These studies highlight that matrix modification is a critical variable in method development for urinary volatilomics. Yet, systematic comparisons of matrix conditions in the context of non-targeted analysis remain limited. since methods vary in sample size, sample preparation, fiber coating, and the analytical devices [4], effecting the extraction of urinary VOCs.

For detection and identification of VOCs, HS–SPME is commonly coupled with gas chromatography–mass spectrometry (GC–MS), which separates and identifies VOCs based on their physicochemical properties such as retention index (RI) and characteristic mass spectra [15]. Early work by Mills and Walker established the feasibility of HS–SPME GC–MS profiling of urinary VOCs using Carboxen/polydimethylsiloxane (CAR/PDMS) fibers and demonstrated increased sensitivity for very volatile and sulphur-containing compounds compared with other coatings [16]. A detailed protocol for urinary VOC metabolomics was later published by Zhang and Raftery [17]. Nevertheless, non-targeted workflows face additional challenges, including incomplete spectral libraries, missing RI values, and variable use of internal standards [18]. Isotopically labeled analogues of individual analytes or compound classes are typically added early in the workflow to support quantitative accuracy. In contrast, internal standards are not routinely employed for peak-area normalization in non-targeted volatilomics, and several methodological studies omit them entirely when the objective is broad profiling rather than absolute quantification [18,19]. Moreover, because compounds differ markedly in their partitioning behavior into the HS, a single internal standard representing only one chemical class may not correct extraction variability across the volatilome and could even bias apparent responses if it is not extracted in a manner comparable to the majority of analytes. Overall, these limitations complicate absolute quantification, but HS–SPME GC–MS remains the most versatile approach for capturing broad VOC profiles.

Recent biological studies also emphasize the clinical potential of urinary VOCs. Trained dogs have demonstrated that urine carries disease-specific volatile signatures and were able to identify acute COVID-19 infection from urine samples, and preliminary findings suggest that detection of post-COVID syndrome (PCS) may also be possible [20]. These results highlight the need for reproducible chemical workflows to complement biological detection.

Inspired by these findings we set out to establish an HS–SPME GC–MS fused strategy tailored for urinary VOC biomarker discovery. Several methods for urinary VOCs have been reported, both for biomarker discovery and for rapid screening [12,21,22]. However, most protocols either require larger urine volumes (2–5 mL), employ different instrumentation (e.g., GC coupled to ion mobility spectrometry), or focus on disease-specific panels. We put a particular focus on a non-targeted volatilomics approach, aiming to maximize the detectable chemical space rather than targeting specific metabolites and optimized the workflow for very small sample volumes (0.75 mL), addressing a key constraint when working with biobank material or rare clinical samples. Within this framework, we systematically investigated the impact of pH adjustment and NaCl addition for ionic strength on extraction efficiency and validated the reproducibility of the method in human urine samples. As a complement to other studies in this field, our work provides a methodological foundation for urinary VOC analysis in clinical biomarker research, particularly under conditions where sample availability is limited.

## 2. Materials and Methods

### 2.1. SPME Conditions and GC–MS Measurement

Urine samples were collected from healthy volunteers and stored at −20 °C until analysis. For SPME measurements, 0.75 mL of urine was transferred into 10 mL glass vials (Ziemer Chromatographie, Langerwehe, Germany). The pH was adjusted to 2–3 using 5 M HCl (Carl Roth GmbH + Co. KG, Karlsruhe, Germany), and 250 µL of a saturated NaCl solution (Carl Roth GmbH + Co. KG, Karlsruhe, Germany) was added. To assess potential contamination from laboratory air and equipment, HCl, or NaCl, blank samples were prepared using Milli-Q H_2_O (mP water purifier) instead of urine, following the same handling procedure. Standards (listed in Table 1 and described in Section 3.4) were prepared in a similar manner, by diluting the compounds in water containing saturated NaCl (pH 2–3) to a final volume of 1 mL.

Extraction temperature and time were fixed, based on commonly applied conditions in previous urinary HS–SPME GC–MS methods and pilot experiments balancing extraction efficiency and sample throughput. Before fiber exposure, samples were incubated at 68 °C for 9 min in an agitator set to 250 rpm. The SPME fiber (75 µm Carboxen/Polydimethylsiloxan (CAR/PDMS), Gerstel, Mülheim an der Ruhr, Germany) was then exposed to the HS of the sample for 24 min at 68°C, while remaining in the agitator at 250 rpm. After extraction, the fiber was thermally desorbed in the GC injector at 250 °C for 4 min in splitless mode.

VOCs were analyzed using an Agilent 7890A GC system coupled to an Agilent 5977B MSD (Agilent Technologies, Santa Clara, CA, USA). Separation was achieved with a Zebron-WAX column (Phenomenex, Torrance, CA, USA, 30 m × 0.25 mm ID, 1.00 µm film thickness). The GC oven program was as follows: initial temperature, 40 °C (10 min hold); ramp at 10 °C/min to 240 °C (5 min hold). Helium was used as carrier gas at a constant flow rate of 1 mL/min. The MS was operated in electron ionization mode (70 eV), with the ion source at 230 °C and the quadrupole at 150 °C. Between runs, the fiber was baked out at 250 °C for 4 min to prevent cross-contamination.

### 2.2. Library Building and Data Processing

GC–MS Chromatograms were processed using Metabolite Detector (MD; Version 3.4202502501) [23]. Prior to sample measurements, a standard mixture of C_10_–C_22_ alkanes was analyzed to enable RI calibration. For peak detection, we applied the new gaussian-matched peak detector.

Final batch quantification was carried out in MD, and the processed data were exported as CSV files for statistical analysis and graphical visualization in R (R version 4.5.1 (13 June 2025)). Error bars in figures represent the standard error of the mean (SEM), and statistical significance is indicated as follows: * *p* < 0.05, ** *p* < 0.01, *** *p* < 0.001.

## 3. Results

### 3.1. Sample Preparation and Method Validation

**Figure 1:**
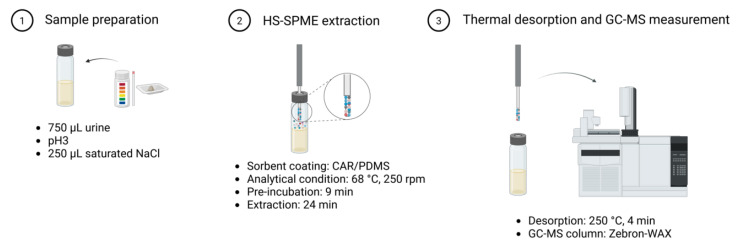
Headspace solid-phase microextraction (HS–SPME) procedure. HS–SPME extraction coupled with GC–MS measurement procedure. Process includes (1) sample preparation (acidification and salt addition), (2) analyte extraction including pre-incubation of the sample, and (3) thermal desorption into the GC inlet, and the GC–MS run for analyte separation, ionization, and detection based on *m*/*z*. Figure created in BioRender.com.

The optimized HS–SPME GC–MS workflow (Figure 1) consisted of adjusting 750 µL of urine to pH 3, adding 250 µL of saturated NaCl (final volume 1 mL), and pre-incubating the sample at 68 °C for 9 min under agitation (250 rpm). VOCs were extracted using a 75 µm CAR/PDMS fiber for 24 min under the same temperature and agitation conditions, followed by thermal desorption in splitless mode at 250 °C for 4 min. Analytes were separated by GC, ionized via electron ionization (70 eV), and detected by MS. Between runs, the fiber was baked at 250 °C for 4 min to prevent carryover.

Matrix modification is a key factor influencing VOC extraction efficiency. We systematically evaluated the effects of pH adjustment and NaCl addition on extraction performance (Table 2), using untreated urine (pH 8) as the baseline. To maintain constant volumes across all conditions, samples without NaCl received 250 µL of water. Blank samples prepared with water instead of urine were analyzed under identical conditions to assess background contamination.

### 3.2. Effect of Matrix Modification on VOC Extraction

**Figure 2:**
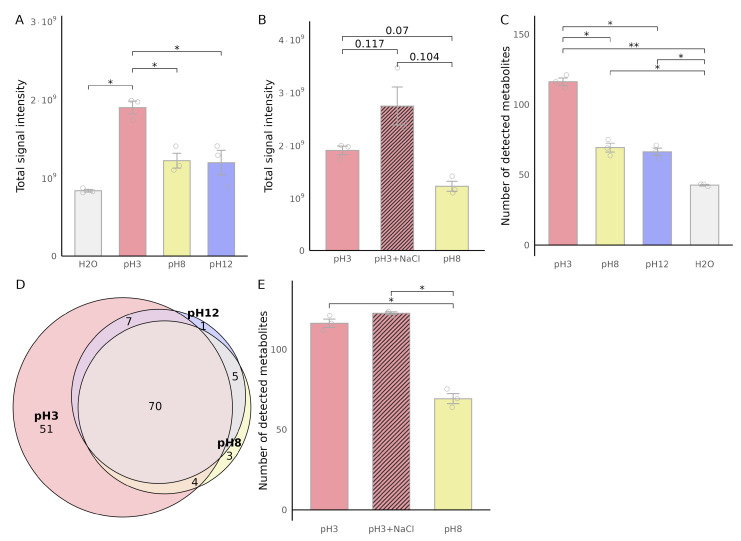
Effect of sample matrix on urinary VOC extraction efficiency. Urine samples (750 µL) were analyzed under different pH and dilution conditions, adjusted to pH 3, pH 8 (untreated urine), pH 12, or diluted with water or NaCl to a final volume of 1 mL. (**A**) Total VOC signal intensity under different pH conditions. (**B**) Effect of NaCl addition on total VOC signal intensity compared to pH 8. (**C**) Number of detected VOCs under different pH conditions. (**D**) Overlap of detected VOCs between the different matrices, with colors indicating urine matrix conditions (pH 3, pH 8, and pH 12). (**E**) Effect of NaCl addition on the number of detected VOCs compared to pH 8. Data are presented as mean ± SEM (n=3). Statistical significance was determined using paired, two-tailed *t*-tests with Benjamini-Hochberg correction (* *p* < 0.05, ** *p* < 0.01).

Chromatographic comparison between naive urine (pH 8) and samples adjusted to acidic (pH 3) or alkaline (pH 12) conditions revealed notable differences in VOC extraction efficiency (Figure 2). Acidic adjustment significantly enhanced VOC extraction, as indicated by an increased total area under the curve (AUC) of integrated chromatographic peaks (Figure 2A). In contrast, alkaline adjustment (pH 12) did not notably alter the total AUC, suggesting that acidification promotes superior extraction efficiency compared to alkaline conditions. Furthermore, combining NaCl addition with an acidic environment (pH 3) resulted in a trend towards an even greater increase in extraction efficiency, suggesting a synergistic effect between acidity and the salting-out phenomenon (Figure 2B), although this effect did not reach statistical significance.

The number of detected chromatographic VOCs mirrored these trends: acidified samples yielded the highest metabolite counts, significantly exceeding both untreated and alkaline conditions (Figure 2C). VOCs overlap analysis revealed that most VOCs detected in acidic samples were also present under other conditions, but 51 VOCs were unique to the acidic matrix (Figure 2D). NaCl addition to acidified samples did not significantly change the total number of detected VOCs (Figure 2E).

Blank chromatograms contained only a small number of low-intensity peaks, attributable to instrumental background, fiber material, or trace contaminants, and were clearly distinguishable from the complex chromatographic profiles of urine samples.

### 3.3. Comparison of Urine Samples and Blanks

**Figure 3:**
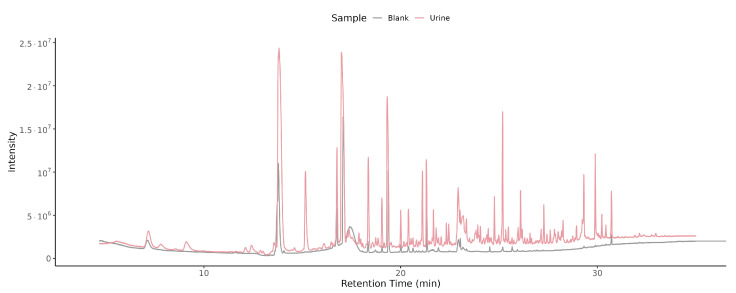
Overlayed TIC of blank and urine samples. Overlay of TICs from a blank injection (grey trace) and a urine sample measured at pH 3 with NaCl (red trace). Retention time (min) is shown on the x-axis and detector intensity on the y-axis. The urine trace contains numerous high-intensity peaks absent in the blank, indicating genuine metabolite signals above background.

Overlaying the total ion chromatograms (TICs) from a blank and a pH 3 + NaCl urine sample (Figure 3) demonstrates the large increase in both number and intensity of peaks in the urine chromatogram. This confirms that the optimized protocol effectively discriminates true urinary VOCs from background noise.

In summary, acidification to pH 3 substantially improves urinary VOC extraction in HS–SPME GC–MS analysis, increasing both total signal intensity and feature diversity. The detection of unique VOCs under acidic conditions highlights the importance of pH adjustment in non-targeted urinary volatilomics workflows.

### 3.4. Evaluation of Individual Standards

To evaluate extraction efficiency and the potential for quantitative analysis, we measured a set of individual VOC standards at three concentration levels (low: 1.25 µM, medium: 2.5 µM, high: 5 µM). The selected standards represent chemically diverse and biomedically relevant VOC classes, including short-chain fatty acids (SCFAs; acetic acid, propionic acid, butyric acid, isobutyric acid, and valeric acid), aromatic compounds (e.g., *o*-cresol), and alcohols (e.g., 1-butanol, 2-propanol). Many of these compounds have been previously reported in urine or other biofluids and are associated with metabolic, renal, or gastrointestinal disorders, ensuring that method performance was assessed across physiochemically distinct and biologically relevant VOCs.

**Figure 4:**
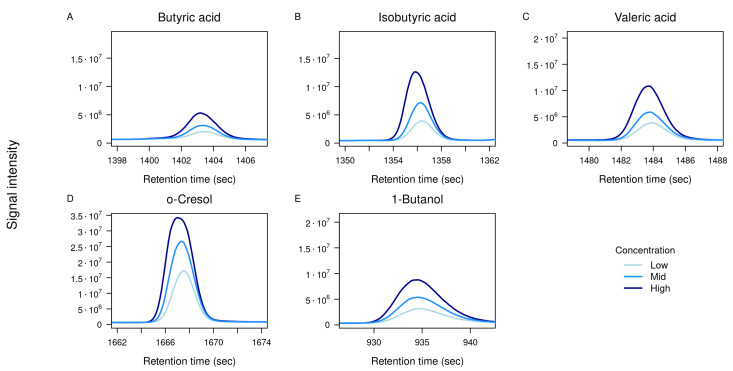
Overlayed TICs of standards at three concentrations. TICs of five selected standards measured at three concentration levels: low (1.25 µM, light blue), mid (2.5 µM, blue), and high (5 µM, dark blue). Panels show zoomed-in retention time windows for: (**A**) Butyric acid, (**B**) isobutyric acid, (**C**) valeric acid, (**D**) *o*-cresol, and (**E**) 1-butanol. The x-axis represents retention time (s), and the y-axis shows detector intensity.

Each compound was analyzed individually at the three concentration levels as listed above. For most standards, a clear concentration-dependent increase in signal intensity was observed, as illustrated by the overlayed TICs in Figure 4, confirming the method’s ability to detect abundance differences across a relevant dynamic range.

**Figure A1:**
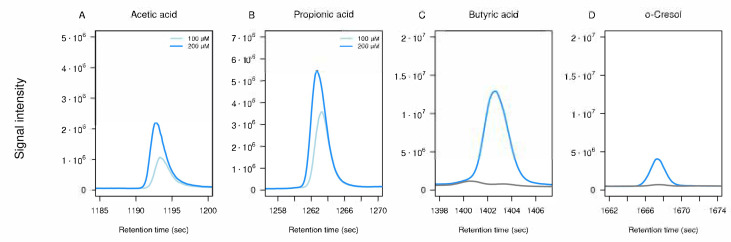
Concentration tests and carry-over assessment for selected standards. Panels (**A**,**B**) show follow-up experiments in which (**A**) acetic acid and (**B**) propionic acid standards were measured at increasing concentrations (100 and 200 µM) to examine peak shape and detector response. Panels (**C**,**D**) show TICs from carry-over experiments in which mid concentrations of (**C**) butyric acid (12.5 µM) and (**D**) *o*-cresol (0.25 µM) were injected, followed by water injections (grey line) to test for residual signal. All panels display zoomed-in retention time windows; the x-axis represents retention time (seconds) and the y-axis shows signal intensity.

Three compounds (acetic acid, propionic acid, and 2-propanol) were not reliably detected at physiological/low µM levels under the applied conditions. However, acetic acid and propionic acid were successfully detected at supra-physiological concentrations of 100–200 µM (Figure A1A,B). Their intrinsically low HS partitioning, due to high water solubility and strong hydrogen bonding, limits detection under these HS–SPME conditions. Alternative fiber coatings or modified extraction parameters could improve sensitivity for such highly water-soluble analytes if targeted quantification is required.

**Figure 5:**
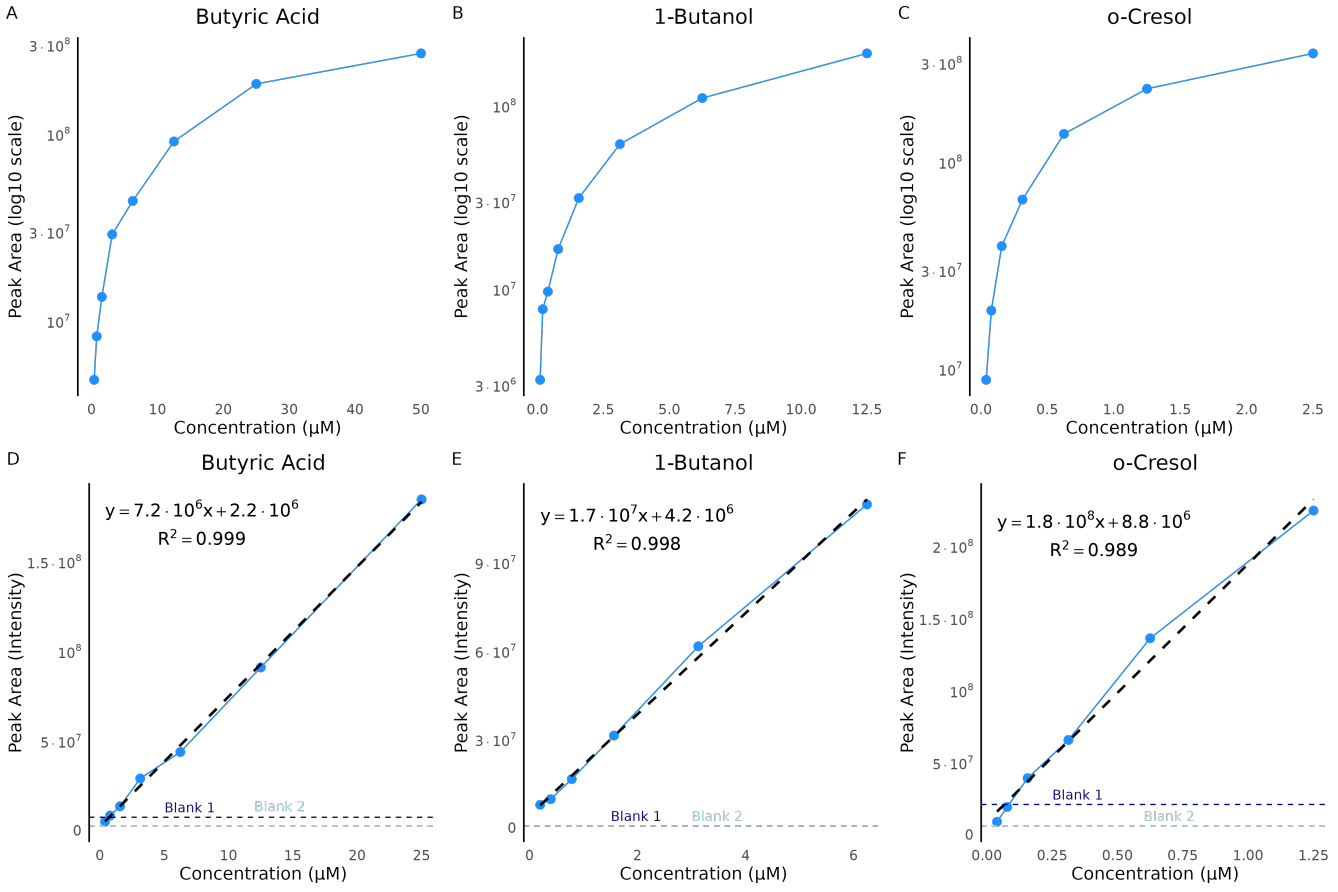
Concentration curves for butyric acid, 1-butanol, and *o*-cresol. Concentration curves were generated from serial dilutions of three selected standards: butyric acid, 1-butanol, and *o*-cresol. Panels (**A**–**C**) show the full concentration range plotted on a log_10_-scaled y-axis: (**A**) butyric acid, 0.39–50 µM; (**B**) 1-butanol, 0.0977–12.5 µM; and (**C**) *o*-cresol, 0.0391–2.5 µM. Panels (**D**–**F**) present the corresponding linear dynamic ranges with raw intensity values, fitted regression lines, equations, and coefficients of determination ($R^{2}$): (**D**) butyric acid, up to 25 µM; (**E**) 1-butanol, up to 0.625 µM; and (**F**) *o*-cresol, up to 1.25 µM. Dashed horizontal lines indicate the signal intensities of blank samples. Each point represents the mean peak area for the respective concentration.

To further evaluate the extraction and quantification efficiency, we measured a series of concentrations of individual standards, covering different chemical classes (Figure 5). Over the full dynamic range, the peak area of butyric acid, a SCFA, increased steadily with concentration up to 25 µM, after which it began to plateau, indicating potential detector or extraction saturation (Figure 5A). For 1-butanol, an alcohol, saturation occurred earlier, around 12.5 µM (Figure 5B). The extraction range for *o*-cresol, an aromatic compound, was shifted toward lower concentrations, with saturation reached at approximately 1.25 µM (Figure 5C).

Notably, the highest quantification ion for butyric acid (50 µM) and *o*-cresol (2.5 µM) exceeded the detector’s dynamic range and showed plateauing in the single ion chromatogram, preventing proper peak integration. However, the linear part of the response curves, indicates the reliable quantification range (Figure 5D–F). These fits exhibited excellent correlation, with $R^{2}$ values of 0.999 for all three compounds, highlighting the importance of defining the linear dynamic range to avoid overestimation caused by saturation effects.

We also investigated potential carry-over effects following injections of highly concentrated standards. To assess this, blank samples were analyzed immediately after the highest concentration injections, as indicated by the dashed lines in Figure 5D–F. For butyric acid and *o*-cresol, these blanks showed residual signal intensities, suggesting minimal carry-over into subsequent runs. Although the butyric acid peaks were not distinct enough to be classified as true detections, the elevated baseline indicated background noise that should be considered. Such high concentrations are unlikely in real urine samples; nonetheless, we also tested for carry-over following mid-level standard injections and observed no detectable carry-over into subsequent blanks (Figure A1C,D).

**Figure 6:**
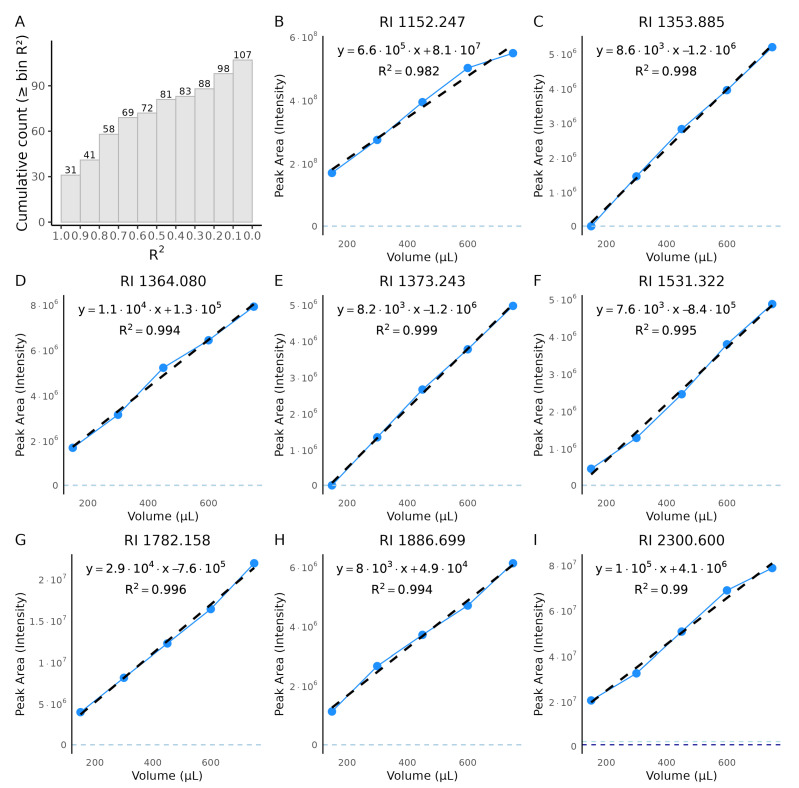
Urine dilution series from non-targeted profiling. Urine volumes ranged from 150–750 µL. (**A**) Cumulative histogram of per-metabolite coefficients of determination ($R^{2}$) from ordinary least-squares fits of peak area versus urine volume (bin width = 0.10 in $R^{2}$ units). Bars (and labels) show the cumulative number of metabolites with $R^{2}$ larger than or equal to each bin; the y-axis reports cumulative counts (*n* = 107 after filtering). Metabolites were retained only if detected at all urine dilutions and if signals at 750, 600, and 450 µL exceeded both blanks. (**B**–**I**) Representative calibration plots for eight non-targeted metabolites; the RI for each metabolite is given in the panel title. Blue points and connecting lines show measured intensities across dilution levels, and black dashed lines are linear fits. Horizontal dashed lines indicate blank intensities. Only metabolites with strong linearity ($R^{2}>0.98$) are displayed. The chemical structures of these non-targeted metabolites are unknown; therefore we report RI.

To evaluate performance in a real biological matrix and assess concentration dependence, we performed a urine dilution experiment using different urine volumes (150–750 µL) while keeping the total sample volume constant at 1 mL. The cumulative distribution of coefficients of determination ($R^{2}$) across 107 detected metabolites (after filtering) is summarized in Figure 6A. Most metabolites exhibited substantial linearity, with nearly half showing $R^{2}$ values above 0.8 and about one-third approaching unity, indicating robust concentration–response behavior across a wide range of compounds. A subset of metabolites yielded lower $R^{2}$ values (<0.6), which likely represent low-abundance or matrix-affected VOCs whose signals approached blank levels or were influenced by non-linear matrix effects. Overall, the median $R^{2}$ of 0.73 reflects consistent concentration dependence for the majority of detected metabolites, confirming that the extraction efficiency scales proportionally with analyte abundance within the tested range.

Representative dilution series are shown in Figure 6B–I. Most metabolites display near-linear responses, confirming that the method captures abundance changes in a complex matrix. Some metabolites deviate from linearity; for example, in Figure 6I, the signal plateaus at the highest volume, consistent with extraction/detection saturation, whereas in Figure 6F, the lowest volume falls outside the reliable linear range. Because these data arise from non-targeted profiling, chemical structures are unknown and the RI is reported for identification.

In summary, concentration-dependent experiments with both chemically diverse VOC standards and urine dilutions confirmed that the optimized HS–SPME GC–MS method reliably detects signal changes within defined linear ranges. Quantitative performance varied by chemical class and compound, with SCFAs, alcohols, and aromatics showing distinct saturation thresholds. Highly volatile analytes such as acetic acid, propionic acid, and 2-propanol were not consistently detected, indicating limitations of the selected fiber coating. In the urine dilution series, most metabolites displayed linear responses across the tested range, although some saturated at higher concentrations while others fell below the linear range at low concentrations. Carry-over was negligible at concentrations relevant to biological samples, supporting the robustness of the method for quantitative urinary VOC analysis.

### 3.5. Application to Biological Samples

To evaluate the applicability of the optimized method to biological urine samples, morning urine was collected from three volunteers on three different days. Samples were stored frozen to mimic typical biobanking conditions and analyzed in technical triplicates. In addition, pooled quality control (QC) samples, prepared by combining equal volumes of all urine samples, were included to monitor potential batch effects and instrument drift. To avoid injecting replicates of the same sample consecutively and thus create batch effects, we acquired the data in blocks: first all samples of replicate 1, followed by all samples of replicate 2, and finally all samples of replicate 3. Pooled QC samples and blanks were interspersed within the sequence.

**Figure 7:**
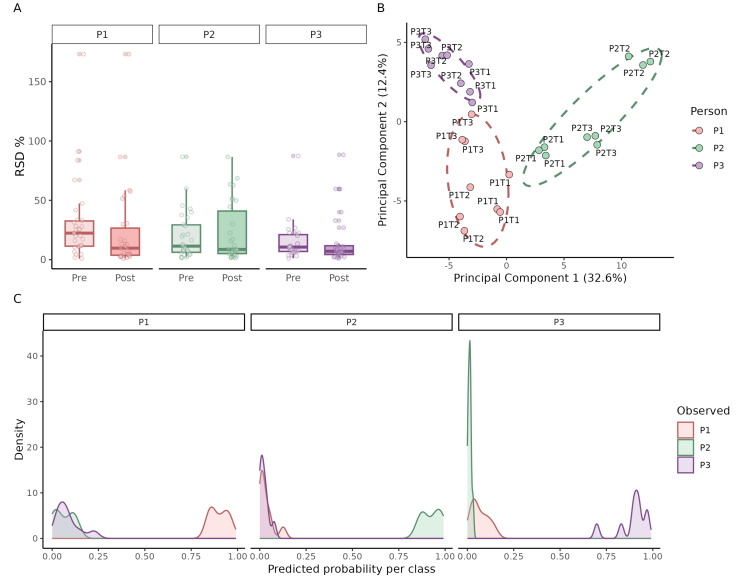
Method performance in real urine samples. Application of the optimized HS–SPME GC–MS method to morning urine samples collected from three individuals (P1–P3) on three different days (T1–T3), each measured in technical triplicates. (**A**) Relative standard deviation (RSD) of the ten most intense VOCs within each triplicate, grouped by individual (Pre = light; Post = dark). (**B**) Principal component analysis (PCA) of all measurements, showing tight clustering of technical replicates and clear separation by individual and collection day. (**C**) Density plots of predicted class probabilities from a Random Forest model, illustrating the ability to correctly assign urine samples to the corresponding individual.

Method performance was assessed by calculating the relative standard deviation (RSD) of the ten most intense VOCs within each triplicate (Figure 7A). For most samples, the RSD was below 25%, demonstrating high analytical repeatability in a biological matrix. After QC-based spline normalization [24], the within-triplicate variation decreased further (median post/pre RSD ratio = 0.72; 95% CI 0.50–1.00), with 60% of VOC per patient showing lower RSD. A paired Wilcoxon test on per-patient median RSD confirmed this reduction (*p* = 0.019). Overall the normalization likely corrected instrument drift typical of longer GC–MS sequences.

Principal component analysis (PCA) (Figure 7B) confirmed tight clustering of technical replicates and revealed clear separation between individuals, as well as day-to-day variation within each person’s VOC profile. Finally, to assess predictive potential, a Random Forest classifier with leave-one-out cross-validation was applied. The predicted probability distributions (Figure 7C) showed that samples were correctly assigned to the corresponding individual with high confidence, underscoring both the reproducibility and discriminative power of the method.

In summary, the optimized method yields reproducible urinary VOC profiles with low intra-sample variability. It reliably detects both inter-individual differences and intra-individual day-to-day variation, while spline normalization further improves stability by reducing replicate RSD. Moreover, Random Forest classification demonstrated that the VOC profiles carry sufficient discriminatory power to accurately predict sample origin at the individual level.

## 4. Discussion

This study demonstrates that non-targeted urinary volatilomics can be performed using very small sample volumes (0.75 mL), while still achieving broad compound coverage and reproducibility. Such an approach is highly relevant for biobank samples and clinical studies, where sample availability is often limited. A key methodological finding is that adapting the urine matrix to an acidic environment markedly improved VOC extraction, both in terms of total signal intensity and number of extracted metabolites. The addition of NaCl showed a trend toward further increasing extraction, suggesting a possible synergistic effect with acidification, although this was not statistically significant. Considering the non-targeted design of the method, maximizing extraction efficiency is particularly important to ensure broad metabolite coverage and thus to increase the likelihood of biomarker discovery.

As reported in previous studies, matrix modification is one of the most important steps in volatilomics method development [2,3]. In particular, acidification improved extraction efficiency not only by increasing the total AUC but also by increasing the number of detectable VOCs. In contrast, alkaline adjustment (pH 12) did not improve extraction compared with untreated urine. Since only analytes in their neutral state can partition efficiently into the HS [25], the enhanced extraction at low pH likely reflects protonation-dependent changes in volatility and partitioning of urinary metabolites. Although the CAR/PDMS fiber used here has high affinity for small, volatile, and often basic compounds, the lack of improvement at alkaline pH suggests that few relevant urinary metabolites are present in a protonated/basic state under these conditions. The observed lack of effect from NaCl addition further indicates that salting-out may play a minor role under our conditions, although other studies have reported stronger effects depending on the sample type and fiber choice [13,26]. Several factors may contribute to this finding. First, with the small urine volume used and the already complex, salty matrix, the relative change in ionic strength may be modest. Second, a combination of slightly increased salting-out, changes in the phase ratio, increased viscosity, and analyte-specific behavior can easily lead to small, compound-dependent effects that cancel out when global metrics such as total peak area are considered [13,18]. Future optimization could therefore explore different ionic strengths or adding salt directly to the sample instead of saturated NaCl.

Beyond ionic strength, other extraction parameters such as temperature and time and fiber coating can also influence HS–SPME performance. In this study, these variables were intentionally kept constant based on widely used HS–SPME protocols and preliminary pilot work showing robust performance under our conditions. Fiber coating is a particularly important determinant of selectivity. Among the coatings commonly used in non-targeted volatilomics, PDMS, CAR/PDMS and DVB/CAR/PDMS differ in their affinity toward specific VOC classes (Table 3), with CAR/PDMS exhibiting the broadest reported compound coverage (227 compounds), followed by DVB/CAR/PDMS (176 compounds) [18]. Therefore, CAR/PDMS was selected as a broadly applicable coating that provides strong performance for the volatile and low-molecular-weight VOCs typically captured in urinary HS–SPME workflows. Once specific VOCs or chemical classes have been identified as biologically relevant targets, the workflow can be refined in a targeted manner. For example through optimization of extraction temperature or time, evaluation of alternative fiber coatings, or the inclusion of isotopically labeled analogues for accurate quantification and process-control monitoring. For applications requiring absolute or semi-quantitative measurements, such isotopically labeled standards are ideally added early in the analytical workflow. Preferably during sample preparation, and, where feasible, even at the point of collection, to correct for losses and variability throughout the extraction and measurement process.

Our serial dilution experiments confirmed compound-specific extraction behavior. Butyric acid, 1-butanol, and *o*-cresol all showed clear concentration-dependent extraction up to a saturation threshold, beyond which peak areas plateaued. This emphasizes the importance of defining linear dynamic ranges to ensure accurate quantification, as overestimation may occur when detector or fiber saturation is reached. For example, butyric acid was linear up to 25 µM, 1-butanol up to 12.5 µM, and *o*-cresol up to 1.25 µM. Carry-over was negligible at concentrations relevant to biological urine samples, although residual signal was detected at very high concentrations, indicating that extended fiber bake-out may be required under extreme conditions. The non-detection of acetic acid, propionic acid, and 2-propanol at physiological/low µM levels (1.25–5 µM) is attributable to their intrinsically low HS partitioning compared to longer-chain analogs like butyric, isobutyric, and valeric acids, or more hydrophobic compounds like 1-butanol and *o*-cresol. These C2–C3 compounds exhibit much higher water solubility and stronger hydrogen bonding (10–100× lower volatility from Henry’s law data), yielding HS concentrations below typical CAR/PDMS HS–SPME LODs despite acidification (pH 2–3) and salting-out that succeed for the others [28,29,30]. Subsequent experiments however confirmed detection of acetic and propionic acids at supra-physiological concentrations (100–200 µM), experimentally confirming their distinctly lower partitioning behavior into the HS compared to the other successfully detected analytes.

Blank measurements revealed a small number of low-intensity peaks, most likely originating from laboratory air, reagents (HCl, NaCl), or fiber background. These VOCs highlight the necessity of careful blank subtraction during biomarker discovery, and only peaks significantly above blank levels should be considered as candidate VOCs. Nevertheless, the chromatographic contrast between blanks and urine samples demonstrated that genuine urinary VOCs can be clearly distinguished, underlining the method’s sensitivity and specificity.

Application of the method to real urine samples confirmed its reproducibility and biological applicability. Triplicate analyses clustered tightly in PCA, and the majority of VOCs showed RSD values below 25%, consistent with common quality criteria in non-targeted metabolomics. Distinct separation between individuals was observed, as well as intra-individual day-to-day variation. Since volunteers received no restrictions regarding diet or lifestyle, this variation likely reflects biological factors such as hydration, dietary intake, or circadian effects. Such natural variability must be considered in biomarker studies, as it can confound disease-specific signals. The inclusion of pooled QC samples, prepared by combining equal volumes of all urine samples, provides an effective tool for monitoring batch effects and instrument drift in future large-scale applications, since a spline normalization to the QC samples reduced the RSD within the replicated per detected metabolite.

To further explore the discriminatory potential of the urinary VOC profiles, we applied a Random Forest classification model with leave-one-out cross-validation. Despite the small dataset and the inherent risk of overfitting, the model was able to assign samples to the correct individual with high predicted probabilities. While these results should be interpreted cautiously, they suggest that the method produces sufficiently rich and consistent VOC signatures to enable statistical discrimination between individuals and to characterize inter-individual metabolic variability in exploratory studies. These insights provide a foundation for planning future clinical studies, including the estimation of technical and biological variability and the prioritization of candidate VOCs for targeted follow-up analyses.

## 5. Conclusions

The optimized HS–SPME GC–MS workflow enables reproducible, non-targeted profiling of urinary VOCs from very small sample volumes. The method provides broad metabolite coverage, robust analytical performance, and applicability to real urine samples, making it well suited for exploratory volatilomics studies under sample-limited conditions. While limitations remain, such as reduced sensitivity for highly volatile compounds and modest salting-out effects under the tested conditions, the workflow establishes a stable analytical basis for future investigations of urinary VOC patterns and supports subsequent targeted method refinement once specific compounds or chemical classes have been prioritized.

## Figures and Tables

**Table 1:** Chemical standards used for HS–SPME GC–MS method development.

Compound	CAS No.	Supplier	Analytical Grade (%)
Acetic acid	64-19-7	Honeywell	≥99.8
Propionic acid	79-09-4	Fluka (Merck)	49–51
Butyric acid	107-92-6	Sigma–Aldrich (Merck)	≥99.0
Isobutyric acid	79-31-2	Fluka (Merck)	≥99.0
Valeric acid	109-52-4	Sigma–Aldrich (Merck)	≥99.0
*o*– Cresol	95-48-7	Sigma–Aldrich (Merck)	≥99.0
2–Propanol	67-63-0	VWR Chemicals	≥99.9
1–Butanol	71-36-3	Sigma–Aldrich (Merck)	≥99.5

**Table 2:** Experimental conditions for urine VOC extraction optimization.

Condition	pH Adjustment	Saturated NaCl Addition
1	untreated (pH 8)	none
2	adjusted to pH 3	none
3	adjusted to pH 12	none
4	untreated (pH 8)	250 µL
5	adjusted to pH 3	250 µL

**Table 3:** Comparison of commonly used SPME fiber coatings for non-targeted VOC analysis.

Fiber Coating	Best Suited for [27]
CAR/PDMS	Permanent gases, very volatile and low–MW VOCs (MW 30–225)
DVB/PDMS	Semi–volatile VOCs (C_6_–C_15_), moderate polarity compounds
DVB/CAR/PDMS	Broad range C_2_–C_20_ VOCs; both volatiles and semi–volatiles

## Data Availability

All raw data for all figures and extended data figures are deposited in the repository platform of Technische Universität Braunschweig at https://doi.org/10.24355/dbbs.084-202510230852-0.

## Abbreviations

The following abbreviations are used in this manuscript:

AUC	Area Under The Curve
CAR	Carboxen
DVB	Divinylbenzol
GC	Gas Chromatography
HS	Headspace
MD	Metabolite Detector
MS	Mass Spectrometry
PCA	Principal Component Analysis
PCS	Post COVID Syndrome
PDMS	Polydimethylsiloxan
QC	Quality Control
RI	Retention Index
RSD	Relative Standard Deviation
SCFA	Short–Chain Fatty Acid
SEM	Standard Error of the Mean
SPME	Solid–phase microextraction
VOC	Volatile organic compound
